# Possibility of Breast Cancer Prevention: Use of Soy Isoflavones and Fermented Soy Beverage Produced Using Probiotics

**DOI:** 10.3390/ijms160510907

**Published:** 2015-05-13

**Authors:** Akimitsu Takagi, Mitsuyoshi Kano, Chiaki Kaga

**Affiliations:** 1 Pharmaceutical Research Laboratory, Yakult Central Institute, Tokyo 186-8650, Japan; 2 Food Research Laboratory, Yakult Central Institute, Tokyo 186-8650, Japan; E-Mails: mitsuyoshi-kano@yakult.co.jp (M.K.); chiaki-kaga@yakult.co.jp (C.K.)

**Keywords:** isoflavone, fermented soy milk, breast cancer, probiotics, *L. casei* Shirota

## Abstract

The various beneficial effects of soybeans, which are rich in phytochemicals, have received much attention because of increasing health awareness. Soy milk that has been fermented using lactic acid bacteria has been used to prepare cheese-like products, *tofu* (bean-curd), and yogurt-type products. However, the distinct odor of soybeans has limited the acceptance of such foods, particularly in Western countries. In Japan, while *tofu* and soy milk have long been habitually consumed, the development of novel, palatable food products has not been easy. The unpleasant odor of soy milk and the absorption efficiency for isoflavones can be improved using a recently developed fermented soy milk beverage. Cancer has been the leading cause of death, and breast cancer is the most common malignancy among women. The most common type of breast cancer is estrogen-dependent, and the anti-estrogenic effects of isoflavones are known. The present review focuses on the characteristics of soy milk fermented using probiotics, an epidemiological study examining the incidence of breast cancer and soy isoflavone consumption, and a non-clinical study examining breast cancer prevention using fermented soy milk beverage.

## 1. Soy Milk and Its Active Ingredients

Soy products, such as soy milk, contain abundant functional ingredients, such as soy proteins and isoflavones, and are attractive materials for use as bases in functional foods. Soy milk, a traditional oriental food and the raw material that is used for making *tofu*, is the water extract of soybeans and is presently considered to be a healthy beverage [1,2,3]. Among the active ingredients of soybeans, isoflavones have attracted particular attention. Isoflavones are known to have a structure that is similar to that of female hormones and may exert either an estrogenic effect by binding to estrogen receptors or an anti-estrogenic effect by inhibiting the binding of estrogen to its receptors [4]. Furthermore, isoflavones have also been shown to have other physiological effects (tyrosine kinase-inhibition, anti-angiogenic effects, activation of natural killer (NK) cells, and antioxidant effects, *etc.*) that act independently of estrogen receptors [5,6,7,8]. In postmenopausal women, the incidences of hypercholesterolemia and arteriosclerosis are known to increase sharply, and the risk of osteoporosis also increases because of a decrease in bone mass associated with a decrease in estrogen secretion. In addition, symptoms such as hot flashes, sweating, shoulder stiffness, arthralgia and palpitation occur during menopause because of the hormonal imbalance that occurs at this time. Isoflavones have been shown to be effective against these symptoms because of their pro-estrogenic effects [1,9,10,11,12]. On the other hand, isoflavones are also known to act prophylactically against breast cancer through their anti-estrogenic effect [13,14].

Isoflavones are usually present in the form of glycosides in soybeans and unfermented soy foods [15,16]. After being ingested, isoflavones are hydrolyzed to aglycones and carbohydrates by β-glucosidase and other enzymes in the gastrointestinal tract, particularly in the lower small intestine, and by the actions of intestinal bacteria; these components are then absorbed from the intestinal tract [17]. In addition, daidzein, a type of isoflavone, can be further metabolized to dihydrodaidzein, equol, and *O*-desmethylangolensin by intestinal bacteria. However, large individual differences exist in this conversion ability, and only 30%–50% of all individuals reportedly have the ability to produce equol from isoflavones, depending on ethnicity and lifestyle [18].

## 2. Fermentation of Soy Milk

Yogurt and probiotic beverages made from milk are generally recognized as excellent functional foods. Soy milk is in itself an excellent functional food [19], and soy milk that has been fermented with probiotics is a novel type of soy milk with additional value. The fermentation of soy milk using lactic acid bacteria or bifidobacteria has various merits that vary greatly depending on the type of bacterial strain used for the fermentation. For example, regarding flavor, fermentation by the above bacterial strains not only substantially reduces the amounts of *n*-hexanal and *n*-pentanal, which are responsible for the strong smell of soy milk, but also reduces the amounts of group A saponin glycosides, which have a strong astringent taste, thereby improving the functionality of the food product, compared with unfermented soy milk [20]. Various bacteria can be used as probiotics to produce fermented soy milk beverage, and fermentation with the *Lactobacillus casei* strain Shirota (LcS), the *Lactobacillus mali* strain YIT 0243, or the *Bifidobacterium breve* strain Yakult reportedly improves the flavor and functionality of fermented soy milk beverage [21].

On the other hand, the isoflavones in soy milk also undergo structural changes during bacterial fermentation. Most isoflavones in soy milk are present as glycosides, as described above, but they are converted to aglycones by fermentation [17]. This ability to convert isoflavones to aglycones varies greatly depending on the type of bacteria, and the above bacterial strains, either alone or in combination, are known to convert isoflavones to aglycones efficiently [22]. Therefore, isoflavone absorption is expected to be increased in fermented soy milk beverage (in which the isoflavones are largely converted to aglycones), compared with soy milk.

**Figure 1:**
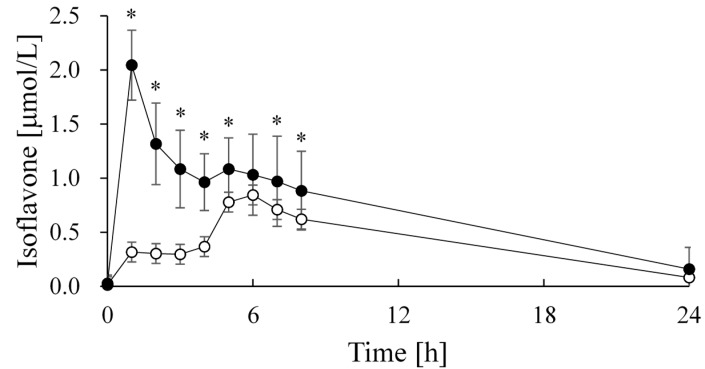
Serum concentration of total isoflavones in healthy adult subjects after the ingestion of soy milk or fermented soy milk beverage [23]. White circle, soy milk; black circle, fermented soy milk beverage. Data were expressed as the mean ± SD (*n* = 12) and were evaluated using a paired *t*-test. * *p* < 0.05. Data from Kano M. *et al.*, *J. Nutr*. **2006** [23].

A study of isoflavone absorption in healthy adults using fermented soy milk beverage produced using the above-mentioned lactic acid bacteria and bifidobacteria revealed that the blood isoflavone levels increased gradually following the intake of soy milk but increased more rapidly following the intake of fermented soy milk beverage (Figure 1). Significant differences in various blood kinetic parameters (*C*_max_, *t*_max_, and AUC) were also observed between soy milk and fermented soy milk beverage (Table 1), suggesting that the fermentation of soy milk likely increases the biological efficiency of isoflavones [23].

**Table 1:** Maximum concentration of serum isoflavones, time taken for the maximum concentration to be reached, and area under the curve for serum isoflavones in healthy adults after the ingestion of soy milk or fermented soy milk beverage [23].

Group	*C*_max_ (μmol/L)	*t*_max_ (h)	AUC (μmol·24 h/L)
Soy milk	0.95 ± 0.38	5.9 ± 1.3	9.55 ± 3.37
FSM beverage	2.04 ± 0.32 *	1.0 ± 0.0 *	17.30 ± 6.38 *

FSM: fermented soy milk. Data were expressed as the mean ± SD (*n* = 12) and were evaluated using a paired *t*-test. * *p* < 0.05. Data from Kano M. *et al.*, *J. Nutr*. **2006** 23].

We have conducted various studies on the above-mentioned fermented soy milk beverage in both animals and humans. The results revealed various effects of fermented soy milk beverage, such as improved lipid metabolism, alcohol metabolism, liver function, bone metabolism, and skin health [24,25,26,27,28]. These effects were also observed using soy milk, but they were more pronounced for the fermented soy milk beverage.

## 3. Breast Cancer

### 3.1. Incidence: Differences between Western and Asian Countries

Breast cancer is the most common malignancy among women, and its incidence and associated mortality rate are increasing worldwide [29]. In particular, its prevalence in Asian countries, which was lower in the past, has been rapidly increasing during the past few decades [30,31]. According to the statistics of the Ministry of Health, Labour and Welfare of Japan, in 2010, malignant neoplasms (cancer) were the most common cause of death (29.5%), followed by heart disease (15.8%) and cerebrovascular disease (10.3%). Furthermore, the lung was the most common organ of malignant neoplasms leading to death in men, followed by the stomach, colorectum and liver, while the colorectum was the most common organ in women, followed by the lung, stomach and breast. An analysis of the annual changes in the major causes of death has revealed that the frequency of malignant neoplasms as a cause of death has steadily increased, and malignant neoplasms have remained the most common cause of death since 1981. At present, malignant neoplasms are responsible for approximately one in three deaths.

A decrease in the incidence of infections because of improved sanitary conditions, the aging of the population, and changing lifestyles, particularly the westernization of diets, have been described as factors associated with the increasing incidence of cancer. The intake of high-fat diets and, in recent years, a lack of exercise have been associated with the increasing incidence of cancer, as exemplified by colorectal cancer, indicating the importance of lifestyle factors in the risk of developing cancer, even though cancer is basically considered a genetic disease. Cancers can affect various organs, and each type of cancer has unique characteristics. Among them, breast, ovarian and prostate cancer are known to be hormone-dependent cancers. In women, breast and ovarian cancers are regarded as hormone-dependent cancers, and their proliferation is dependent on the availability of female hormones. In 1994, the number of new breast cancer cases among Japanese women surpassed that of stomach cancer to become the most common cancer in Japanese women. An estimated 45,700 women were newly diagnosed as having breast cancer in 2003 (whereas an estimated 36,500 were diagnosed as having stomach cancer) [32]. One of the likely causes of the rapid increase in breast cancer in Japan is thought to be increased estrogen exposure, which is an important risk factor [33]. Changes in the traditional Japanese lifestyle and an increase in the incidence of obesity are also possible contributing factors [34].

The number of women who develop breast cancer is more than three times the number of women who die of breast cancer, and this is thought to be related to the relatively high survival rate of women with cancer of the breast. Therefore, an increase in the cancer screening rates for breast cancer is likely to increase early detection, enabling an improved maintenance of quality of life even among patients who are diagnosed as having breast cancer. Based on data collected in 2010, approximately one in every 12 women develops breast cancer, and this incidence is expected to increase further in the future.

Information on the incidence of breast cancer, *etc.*, has revealed problems specific to breast cancer. Statistical data on the incidence of breast cancer classified according to age group in 1975 and thereafter and prepared by the National Cancer Center (Japan) showed two noteworthy features. One was the annual increase in the incidence, and the other was the increase in the incidence between the ages of 30–39 years, with the peak age at onset falling between 40 and 44 years in all the surveyed years. Regarding the onset of cancer, it is understood that gene abnormalities occur in normal cells due to genetic mutations or epigenetic modifications of the DNA sequence, and some of the cells in which these abnormalities are not corrected become cancerous and proliferate to a stage where the number of cancerous cells becomes sufficient to be detected as cancer tissue. Therefore, cancer development is generally thought to take many years. From the fact that the incidence of breast cancer increases from the ages of 30 to 39 years with the peak age at onset falling between 40 and 44 years, it can be speculated that cancer cells in the breast develop at an age younger than 30–39 years. Although we can understand why women aged ≥40 years undergo breast cancer screening in the present system, we think that it is important to pay attention to the age groups before the incidence peak, *i.e.*, adolescence or younger ages, from a prophylactic standpoint.

### 3.2. Risk Factors and Lifestyle

Early menarche, a late age at first birth, late menopause, and a lower number of children, *etc.*, are well-known as risk factors for the development of breast cancer, indicating that exposure to female hormones plays an important role in the development of breast cancer. Namely, an early age at menarche and a late age at menopause may be interpreted as prolonged exposure to female hormones during life. A study of the risk factors involved in cancer development has indicated that obesity, alcohol intake, *etc.* are definite risk factors for the development of breast cancer and that breastfeeding is a definite factor that decreases the risk of breast cancer [35]. In addition, a survey of ethnic groups and residential areas was conducted as part of a study on risk factors for cancer. The incidence of breast cancer among Japanese persons living in Hawaii or San Francisco is reportedly more than twice as high as that in Japanese women living in Japan [36]. An increase in the incidence of breast cancer according to the residential area, even among Japanese women who are considered to have a similar genetic background, suggests that changes in the living environment, particularly dietary habits, are closely involved in the development of breast cancer. The female hormone estrogen is also known to be important for the development of breast cancer. The most common types of breast cancer are estrogen-dependent (approximately 70% of breast cancer patients have estrogen receptor-positive breast cancer), and these breast cancer cells proliferate using estrogen. Estrogen is important for the development of femininity, and at the same time, has the negative aspect of serving as a risk factor for breast cancer. In addition, because estrogen secretion is suppressed during pregnancy, fewer childbirths can be interpreted as serving as a risk factor for the development of breast cancer as a result of the reduced duration in which estrogen activities are suppressed. As described above, female hormones, particularly estrogen, are closely involved in the proliferation of breast cancer cells and can be influenced by lifestyle. Therefore, improvements in lifestyle are recommended to prevent breast cancer. As described in the previous section, isoflavones are expected to have a breast cancer-preventive effect through their anti-estrogenic effect, based on the structural similarity between estrogen and isoflavones.

## 4. Epidemiological Study on Breast Cancer

The peak age of onset among patients with breast cancer is 40 to 44 years in Japan, and the westernization of lifestyle from an even younger age may be involved in the development of breast cancer. An epidemiologic study of breast cancer was conducted in Japan using a lifestyle questionnaire on topics such as dietary habits and exercise from girlhood to present. The subjects were women aged 40 to 55 years and consisted of 306 breast cancer patients (cases) and 662 healthy women without breast cancer (controls); the results of the survey were compared between the breast cancer patients and healthy women (case-control study) [37]. The questionnaire contained an item related to the intake of soy foods, which are expected to reduce the risk of breast cancer. In addition, the intake of dairy products containing LcS, which has been reported to contribute to the prevention of recurrences of bladder cancer and to reduce the risk of colorectal cancer in humans studies [38,39,40,41], was investigated. To ensure the accuracy of the study, the survey was conducted by trained investigators who were blinded to the group assignments. In addition, the subject candidates had a known history of having purchased dairy products, *etc.* The survey revealed that 84.9% of the breast cancer patients who participated in the study had estrogen receptor-positive breast cancer. The odds ratio, which is a statistical measure of the likelihood of developing a disease *etc.*, was 1.00, 0.76, 0.53, and 0.48 for groups with soy isoflavone intakes of <18.76, 18.76–28.81, 28.81–43.75, and >43.75 mg/day, respectively, showing that the risk of breast cancer decreased with an increase in the daily intake of soy isoflavones. On the other hand, the odds ratio was 1.0 and 0.65 for groups drinking dairy products containing LcS <4 times and ≥4 times a week, respectively, indicating that the risk of breast cancer decreased with an increased frequency of drinking these products. Furthermore, daily soy isoflavone intakes of <18.76 and ≥43.75 mg were defined as “low” and “high” intakes, respectively, and the drinking of dairy products containing LcS <4 times and ≥4 times a week were defined as “low” and “high” frequencies, respectively, to analyze the interrelationships among these factors. As a result, the odds ratio was 1.00 in the group with a low frequency of drinking dairy products containing LcS and a low intake of soy isoflavones, 0.50 in the group with a high frequency of drinking dairy products containing LcS and a low intake of soy isoflavones, 0.49 in the group with a low frequency of drinking dairy products containing LcS and a high intake of soy isoflavones, and 0.36 in the group with a high frequency of drinking dairy products containing LcS and a high intake of soy isoflavones, indicating that the risk of breast cancer was lowest when a large amount of both soy isoflavones and dairy products containing LcS were taken. Therefore, dietary habits involving the intake of both soy foods and dairy products containing LcS were suggested to be useful for preventing breast cancer. In addition, the maintenance of these desirable dietary habits since adolescence was considered to be important.

## 5. Non-Clinical Study of Breast Cancer Prevention

Some probiotic strains reportedly release bioactive metabolites through the fermentation of milk, and the released molecules can prevent murine breast tumor growth by stimulating an immune response [42]. However, it is expected that there is little protective efficacy of cancer with these types of bacterial strains in the case of fermentation of soy milk and not all probiotic strains exert cancer preventive effects. In other words, strain specificity is clearly existing in cancer preventive effects [43]. Several studies have been conducted to verify the potential of fermented soy milk beverage to inhibit tumor cell growth, but, the active components that were examined were not phytochemicals [44,45]. Fermenting soy milk using *Streptococcus thermophilus* 14085 or *Bifidibacterium infantis* 14603 reduced the saponins and phytates contents and increased the total phenolic content. The crude extracts obtained from this fermented soy milk beverage exhibited a suppressive effect on the *in vitro* proliferation of the human colon cancer cell lines HT-29 and Caco-2 [44]. In a breast cancer model, fermented soy milk beverage containing *Lactobacillus acidophilus*, *Lactobacillus bulgaricus*, *Streptococcus lactis*, or *Bifidobacteria* inhibited the growth of estrogen-receptor positive MCF-7 human breast cancer in mice, and this effect was mainly involved in the generation of reactive oxygen species [45]. The fermentation of soy milk using *Bifidobacterium breve* increased the content of isoflavone aglycone, and this fermented soy milk beverage inhibited rat mammary carcinogenesis [46]. Although most types of breast cancer exhibit estrogen-dependent growth, studies focusing on the phytochemicals of fermented soy milk beverage are still lacking.

To test the results obtained from the above-mentioned epidemiologic study [20] in a prospective manner, a study was conducted using a chemically induced mammary tumor model [47]. Soy milk alone, LcS alone, or a combination of soy milk and LcS were fed to rats in which breast tumors had been chemically induced using PhIP (2-amino-1-methyl-6-penylimidazo[4,5-b]pyridine). Mammary tumor development was then observed chronologically, and a mammary tumor-suppressing effect was observed in all the experimental diet groups (Table 2). A histopathological analysis of the tumor tissues showed that the tumors induced in this model were estrogen receptor-positive. The proportion of Ki-67-positive cells (which is an indicator of cell proliferation and important for breast cancer classification) and the proportion of estrogen receptor-positive cells were significantly reduced in the combination group. Interestingly, the incidence was reduced in the soy milk group, the tumor volume was reduced in the LcS group, and both were reduced in the combination group. The number of tumors per rat was markedly reduced in the combination group. Therefore, the use of probiotics may complement the cancer prevention profile that cannot be achieved using soy milk alone. In the case of LcS, the enhanced activity of NK cells induced by LcS inhibits murine carcinogenesis [48], and this up-regulation of NK cell activity by LcS has also been demonstrated in healthy humans [49,50,51].

**Table 2:** Incidence, multiplicity, volume and immunohistological profiles of mammary tumors in PhIP (2-amino-1-methyl-6-penylimidazo[4,5-b]pyridine)-exposed rats [47].

Group	Incidence (%) †	Multiplicity (tumors/rat) ‡	Volume (cm^3^/tumor) §	Tumor Tissue Profile	
				ER-α (%)	Ki-67 (%)
Control	73.8 (31/42)	2.7 ± 0.5	1.3 ± 0.2	55.1 ± 1.7	25.9 ± 1.0
Soy milk	59.5 (25/42)	1.6 ± 0.3	2.5 ± 1.1	57.1 ± 1.6	24.5 ± 1.2
LcS	73.8 (31/42)	3.0 ± 0.5	0.8 ± 0.1	60.8 ± 1.5	24.5 ± 1.0
LcS + Soy milk	59.5 (25/42)	1.2 ± 0.2 *	0.8 ± 0.2	47.2 ± 2.0	21.8 ± 1.4

All data were expressed as the mean ± SE. **†** Number of rats with mammary tumors per effective number of rats; **‡** Number of mammary tumors per rat; **§** Tumor volume was calculated using the following formula: tumor volume = (length) × (width) × (height) × π/6. All mammary tumors were immunostained for estrogen receptor-α (ER-α) and Ki-67 and the ratios of ER-α and Ki-67 expressing cells were evaluated. The tumor incidence was analyzed using the χ^2^ test. The tumor multiplicity and volume were analyzed using the Dunnett test relative to the control group. * *p* < 0.05. Data from Kaga C. *et al.* *Cancer Sci*. **2013** [47].

**Figure 2:**
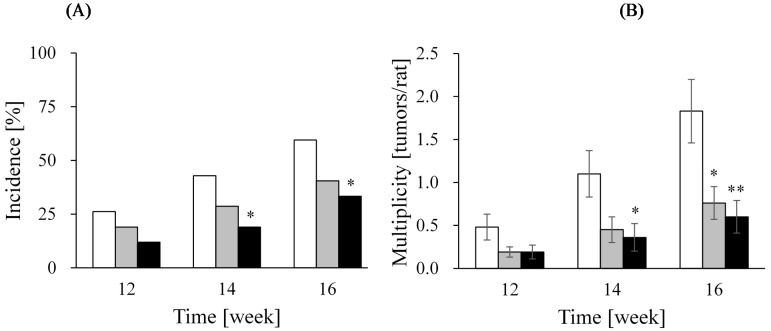

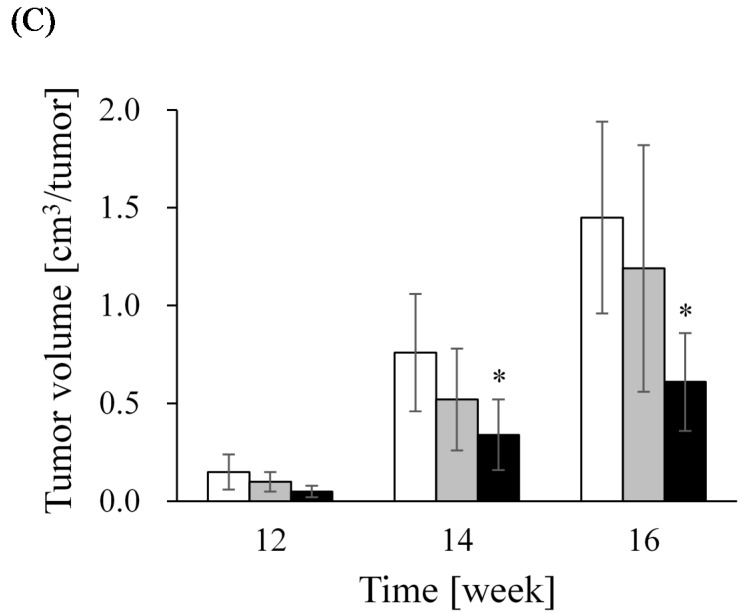
Time course changes in palpable mammary tumors in PhIP-exposed rats in 12th, 14th and 16th week after the start of the experiment. (**A**) Tumor incidence; (**B**) multiplicity; and (**C**) tumor volume. White bar, control; gray bar, soy milk and black bar, fermented soy milk beverage. All the data were expressed as the mean ± SE. The tumor incidence was analyzed using the χ^2^ test. The tumor multiplicity and volume were analyzed using the Dunnett test relative to the control group [52]. * *p* < 0.05; ** *p* < 0.01. Kaga C. *et al.* Unpublished data [52].

The above results suggested that the intake of soy milk and LcS may have a synergistic effect. Therefore, in an attempt to facilitate the intake of both soy milk and LcS and to increase the ratio of soy isoflavone aglycones in soy milk, a fermented soy milk beverage was developed using the probiotic microorganism LcS, as mentioned in a previous section. The breast cancer-preventive effect of this fermented soy milk beverage was tested using a similar PhIP-induced mammary tumor model in rats. Because this experimental model develops tumors in the mammary glands approximately 10 weeks after the start of the experiment, the tumor incidence after the 12th week was monitored. As a result, the incidence of mammary tumors was reduced by soy milk, but the suppression of mammary carcinogenesis was more potent in the fermented soy milk beverage group than in the soy milk group (Figure 2) [52].

In addition to the results of an epidemiological study examining breast cancer prevention, the results of clinical studies have also shown that LcS can exert a cancer preventive effect. Some strains of probiotics have exhibited a cancer preventive efficacy in animal models, but to our knowledge, LcS is the only strain that has been shown to be effective in human studies. A prospective cohort study in patients with previously treated colorectal cancer compared the incidence of the reappearance of colorectal neoplasms during the study period between patients with and those without the intake of LcS [41]. The results indicated that the daily intake of LcS reduced the risk of the recurrence of colorectal cancer. Although no difference was seen in the overall incidence of tumors detected by a colonoscopy after 2 or 4 years, the 2-year incidence of tumors with a diameter ≥4 mm and the 4-year incidence of tumors with moderate or high-grade atypia were significantly lower among the subjects who had consumed LcS. In addition, a multicenter, randomized, controlled study was conducted in patients with superficial bladder cancer. The primary endpoint of this study was the 3-year recurrence-free rate for bladder cancer [40]. The results indicated that the daily intake of LcS reduced the risk of the recurrence of bladder cancer. The 3-year recurrence-free rate was significantly higher among subjects who had taken LcS orally in combination with standard intravesical chemotherapy with epirubicin than in those who received intravesical epirubicin alone (74.6% *vs.* 59.9%). A double-blinded trial was also conducted in 138 patients with superficial transitional cell carcinoma of the bladder following a transurethral resection to evaluate the prophylaxis of recurrence. As a result, the oral administration of an LcS preparation was found to be safe and effective for preventing the recurrence of superficial bladder cancer [38]. In addition, a case-controlled study was also conducted to compare lifestyle factors between Japanese patients with superficial bladder cancer and control subjects [39]. The results suggested that the habitual intake of fermented milk containing LcS could prevent the development of bladder cancer. Therefore, in addition to the physiological effect of soy isoflavones, soy milk fermented with LcS may be capable of enhancing the cancer preventive potency of soy isoflavones as a functional food.

## 6. Concluding Remarks

Analyses of cohort studies, *etc.*, have shown that soybean intake may reduce the risk of breast cancer in Japanese women and have excluded the involvement of isoflavones in the promotion of breast cancer [13,53]. In the WHO-CARDIAC study populations, the lower mortalities of breast cancers were shown to be inversely related to the 24-h urinary isoflavone excretion [54]. A longitudinal prospective study of 5042 breast cancer patients in China revealed that soy food intake is safe and was associated with a lower mortality and recurrence among breast cancer patients [55]. In Western countries, where the incidence of breast cancer is high, the effectiveness of chemoprevention by inhibiting the actions of estrogen using the hormone antagonist tamoxifen, which is classified as a selective estrogen receptor modulator, or other agents has been tested, and such chemoprevention has been approved by the US Food and Drug Administration (FDA) agency. Because the postmenopausal incidence of breast cancer remains almost unchanged in Japan, unlike the situation in the USA, it is considered difficult to apply the results of chemoprevention trials using drugs in the USA, without modification, to Japanese women [56]. On the other hand, interventional studies using dietary modification have been conducted for breast cancer, *etc.* [57,58,59,60]. However, none of the studies conducted so far have shown a clear preventive effect, and the need for dietary modification at younger ages than middle and old age has been pointed out, in addition to issues regarding the establishment of the intervention period and the calculation of the intervention effect. Because factors related to adolescence, such as age at menarche, are recognized as risk factors for breast cancer, dietary modification during middle and old age alone may not have a sufficient cancer risk-reducing effect. The accumulation of further scientific evidence is needed. The utilization of fermented soy milk beverage with probiotics has the potential to be useful as a novel functional food for reducing the risk of breast cancer.
